# No Evidence that 2D:4D is Related to the Number of CAG Repeats in the Androgen Receptor Gene

**DOI:** 10.3389/fendo.2013.00185

**Published:** 2013-12-05

**Authors:** Johannes Hönekopp

**Affiliations:** ^1^Department of Psychology, Northumbria University, Newcastle upon Tyne, UK

**Keywords:** testosterone, 2D:4D, AR gene, CAG repeats, meta-analysis

## Abstract

The length ratio of the second to the fourth digit (2D:4D) is a putative marker of prenatal testosterone (T) effects. The number of CAG repeats (CAGn) in the AR gene is negatively correlated with T sensitivity *in vitro*. Results regarding the relationship between 2D:4D and CAGn are mixed but have featured prominently in arguments for and against the validity of 2D:4D. Here, I present random-effects meta-analyses on 14 relevant samples with altogether 1904 subjects. Results were homogeneous across studies. Even liberal estimates (upper limit of the 95% CI) were close to zero and therefore suggested no substantial relationship of CAGn with either right-hand 2D:4D, left-hand 2D:4D, or the difference between the two. However, closer analysis of the effects of CAGn on T dependent gene activation *in vitro* and of relationships between CAGn and T dependent phenotypic characteristics suggest that normal variability of CAGn has mostly no, very small, or inconsistent effects. Therefore, the lack of a clear association between CAGn and 2D:4D has no negative implications for the latter’s validity as a marker of prenatal T effects.

## Introduction

In contrast to circulating testosterone (T), perinatal T has long-lasting, “organizational” effects in many species, including humans (1). In the latter, T levels are particularly relevant during the second trimester, but their effects are notoriously difficult to study (2). However, 2D:4D (i.e., the length of the second digit divided by the length of the fourth digit) seems to track prenatal steroid effects (3), thereby providing an easily accessible, though probably noisy (4–6) index of individuals’ hormonal past. In short, this is evidenced by experiments in mice [e.g., Ref. (7)]; correlations between steroid levels in amniotic fluid and 2D:4D at age 2 years (8); masculinized (i.e., lowered) 2D:4D in females exposed to high prenatal T levels caused by congenital adrenal hyperplasia [*d* ≈ 0.8; Ref. (9)]; and feminized 2D:4D in (i) genetic males with complete androgen insensitivity syndrome [*d* ≈ 0.5; Ref. (4)] and (ii) in males with Klinefelter’s syndrome [*d* ≈ 0.8; Ref. (10)], a condition associated with low T levels throughout development. The sex difference in 2D:4D seen in adults [*d* ≈ 0.4; Ref. (9)] is established *in utero* [*d* ≈ 0.6; Ref. (11, 12)]; individual 2D:4D scores show stability during development, including puberty [e.g., Ref. (13)] and are unrelated to baseline circulating T levels in adults (14, 15).

Testosterone effects depend on a structure called androgen receptor (AR), which comes in different variants, some of them leading to stronger T effects than others. The relationship between these AR variants and 2D:4D has received considerable attention, based on the notion that if 2D:4D reflects prenatal T effects and if AR variants moderate T effects, AR variants should show systematic relationships with 2D:4D [e.g., Ref. (16, 17)]. The current paper seeks to describe this relationship. However, before this is addressed further, it is necessary to look at the link between the AR and T effects in greater detail.

Testosterone regulates the transcription of genes, and this depends on the AR. In the cytoplasm, the AR is bound to heat-shock proteins and therefore inactive. When binding with T or dihydrotestosterone, the AR sheds its heat-shock proteins, changes into an active shape and migrates to the cell nucleus. There, it connects with coactivators and another AR and then binds in this dimerized form to specific sites in the DNA where it regulates the transcription of target genes (18, 19).

**Figure 1:**
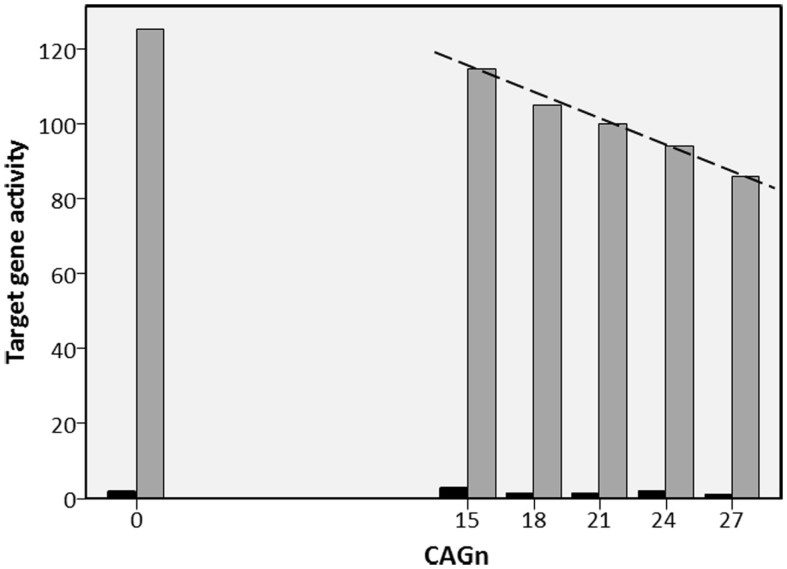
**Fictitious example of *in vitro* study into the effect of CAGn on target gene activity**. Example for regression of the activity of a testosterone regulated target gene on CAGn in *in vitro* studies (cf. Table 1). ARs with CAGn of 0, 15, 18, 21, 24, or 27 are used either with testosterone (gray bars) or without testosterone (black bars). Target gene activity observed at CAGn most typical in humans (21) is set to 100%. Then the regression slope (dashed line, −2.3%) is calculated to describe target gene activity as a function of CAGn. In cases like the present, where CAGn outside the human range produce a deviation from linearity (here 0 CAGn), the regression slope was calculated only for those CAGn that showed a linear function.

The AR is produced by the AR gene, which is located on the X-chromosome. On exon 1, this gene repeats the nucleotide sequence CAG; the number of these repeats (CAGn) varies inter-individually in length and codes for the length of a polyglutamine stretch on the N-terminal domain of the AR. Most humans have CAGn between 15 and 30, the average is about 22 with a standard deviation of about 3.5 (20). Experiments *in vitro* demonstrated that longer polyglutamine stretches make the AR less effective, resulting in less AR-regulated genetic activity [e.g., Ref. (21–23)]. In such studies, cell lines from either monkey kidneys or human prostate cancer are transfected with AR gene variants that differ in CAGn. Subsequent activity of a target gene is then measured in the presence and absence of androgen. How strong is the effect of CAGn on target gene activity? I used figures in relevant reports (20–26) to calculate regression slopes that reflect by what proportion target gene activity drops for each additional CAG repeat (cf. Figure 1); activity at “normal” CAGn (around 20) served as the 100% baseline in each case. Where non-linear effects occurred at CAGn outside the normal human range (20, 25), I restricted the computation of the regression slope to the CAGn range that produced a linear effect. Figure 1 illustrates this process based on a fictitious *in vitro* study. As can be seen from Table 1, which provides an overview of the results, regression slopes averaged −2.3% for cell lines from monkey kidneys and −1.2% for prostate cancer cell lines.

**Table 1:** **Change in androgen driven target gene activity per additional CAG repeat in the AR gene in *in vitro* studies**.

Study	Cell type	CAGn range tested	Change (%)
Beilin et al. (21)	Monkey kidney	15–31	−2.8
Callewaert et al. (24)	Monkey kidney	0–9	−3.9
Chamberlain et al. (22)	Monkey kidney	25–77	−0.7
Kazemi-Esfarjani et al. (26)	Monkey kidney	0–50	−1.9
Beilin et al. (21)	Prostate cancer	15–31	−1.4
Buchanan et al. (20)	Prostate cancer	16–35	−1.8
Ding et al. (23)	Prostate cancer	14–25	−1.0
Irvine et al. (25)	Prostate cancer	9–42	−0.7

In short then, high CAGn is associated with low androgen sensitivity *in vitro*; hence, a positive relationship between CAGn and 2D:4D might be expected. The first report of such a correlation (17) became one of the most frequently cited papers in the 2D:4D literature; however, later studies showed an inconsistent picture with a mixture of positive and negative findings [e.g., Ref. (27, 16)]. The relationship between CAGn and 2D:4D has often played a prominent role in discussions of the validity of 2D:4D as a marker of prenatal T effects. For example Breedlove (5) argued, “the strongest evidence that androgens affect digit ratios is the report (17) that normal polymorphism in the AR gene correlates with digit ratios in men” (p. 4117); conversely, Hampson and Sankar (16) concluded that their failure to find a positive relationship between CAGn and 2D:4D “call[s] into question the widespread assumption that small differences in the size of […] [2D:4D] are an accurate gage of relative differences across individuals in fetal testosterone exposure” (p. 560).

This paper has two purposes. First, to clarify the relationship between 2D:4D and CAGn; to this end, I present a meta-analysis of the relevant literature. And second, to discuss in greater detail the implications of this relationship for the validity of 2D:4D as a marker of prenatal T effects.

## Materials and Methods

Studies were retrieved with the search terms *2D:4D* OR *digit ratio* in conjunction with *CAG* OR *AR* in the *topics* field in *ISI Web of Science* and in the *MeSH Major Topic* field in *PubMed*; this resulted in nine relevant studies from which 14 samples with 792 females and 1331 males entered the analyses. For all samples, CAGn was treated as a continuous measure and I report Pearson correlations with 2D:4D in all cases. As females (but not males) have two AR gene copies, either the shorter allele, the longer allele, or the bi-allelic mean can be used. One report (28) reported all three analyses (which led to very similar results), and I used the result for the bi-allelic mean in the present analysis. For two other reports that involved females (29, 30) it remained unclear on which of the three measures their analysis was based.

In line with the approach in the primary studies, separate meta-analyses were run for 2D:4Dr (right-hand 2D:4D), 2D:4Dl (left-hand 2D:4D), and D_r-l_ (2D:4Dr × 2D:4Dl). One longitudinal study (29) reported multiple results for each 2D:4D measure and CAG repeats in the same sample. These were averaged so that each sample contributed only one effect size in each meta-analysis. The Knickmeyer samples and the Loehlin et al. (30) study contained sib-pairs. Although this creates statistical dependencies, the weighting of these samples in the analyses was not corrected downwards, mostly because it did not matter, as I will discuss later. Typically, samples showed little or no ethnic heterogeneity; for one atypical study (31), results with ethnic group as a covariate were used. Where relevant information was missing in the publications, authors were contacted (cf. note in Table 1). Random-effects meta-analyses were performed (32), which model the population correlation as a random variable with mean ρ and variance τ^2^. Due to chance effects in sampling, multiple studies into the same phenomenon are expected to produce different results, resulting in variance of the correlations in primary studies. If the observed variance exceeds the variance to be expected due to random sampling, this suggests that primary studies differ in a systematic fashion, i.e., that not all tap into the same population correlation. E.g., the correlation between CAGn and 2D:4D might differ for females and males, young and old, etc. τ^2^ reflects to what extent the observed variance in correlations exceeds the variance expected due to random sampling. The *Q*-statistic is used to test if this excess variance deviates significantly from zero. In the results, I report the standard deviation τ instead of the variance τ^2^ because the former is easier to interpret. Analyses were carried out with *Comprehensive Meta-Analysis* (2.2.064).

## Results

The results for individual studies are listed in Table 2. The results of the three meta-analyses are summarized in Table 3. As can be seen from column ρ, estimates for all population correlations were close to (and not significantly different from) zero, and all upper limits of the 95% CI were *r* < 0.09. Estimates for random variance around ρ were zero or small, and not statistically significant. Therefore, no attempt was made to explain differences across study results via meta-regression. Results remained basically unchanged when: the female and mixed-sex samples (*k* = 4) were removed (cf. Table 3); when an unusual sample of male-to-female transsexuals was removed (detailed results not shown here); or when all of the previous were excluded from analysis (detailed results not shown here).

**Table 2:** **Primary studies investigating the relationship between 2D:4D and CAGn**.

Study	Country	Age	Sex	CAGn	*N*	*r*
**2D:4D RIGHT HAND**						
Manning et al. (17)	England	32.6 ± 14.2	M	21.4 ± 2.3	50	0.29*
Butovskaya et al. (27)^a^	Tanzania	≈34 ± 13	M	22.5 ± 2.2	107	0.135
Folland et al. (33)	England	20.1 ± 2.2	M	26 ± 4	77	0.10
Loehlin et al. (30)	Australia	≈14	F		218	0.08
Hurd et al. (31)	Canada	≈19 ± 2	M		155	0.05
Knickmeyer et al. (29)^a^	USA/Asian	≈1	M/F		6	0.04
Zhang et al. (28)	China	19.9 ± 1.4	F		391	0.030
Mas et al. (34)^a^			M		70	0.005
Zhang et al. (28)	China	19.9 ± 1.4	M		294	0.003
Knickmeyer et al. (29)^a^	USA/Black	≈1	M/F		31	− 0.01
Knickmeyer et al. (29)^a^	USA/White	≈1	M/F	≈19.7 ± 2.5	108	−0.04
Mas et al. (34)^a^			M^b^		63	−0.04
Loehlin et al. (30)	Australia	≈14	M	22.1 ± 3.1	182	−0.06
Hampson and Sankar (16)	Canada	18.7 ± 1.6	M		152	− 0.085
**2D:4D LEFT HAND**						
Folland et al. (33)	England	20.1 ± 2.2	M	26 ± 4	77	0.2
Butovskaya et al. (27)^a^	Tanzania	≈34 ± 13	M	22.5 ± 2.2	107	0.191*
Loehlin et al. (30)	Australia	≈14	F		218	0.14*
Zhang et al. (28)	China	19.9 ± 1.4	M		294	0.016
Manning et al. (17)	England	32.6 ± 14.2	M	21.4 ± 2.3	50	0.005
Mas et al. (34)^a^			M		70	− 0.014
Zhang et al. (28)	China	19.9 ± 1.4	F		391	− 0.018
Knickmeyer et al. (29)^a^	USA/White	≈1	M/F	≈19.7 ± 2.5	111	−0.03
Hampson and Sankar (16)	Canada	18.7 ± 1.6	M	22.1 ± 3.1	152	−0.063
Hurd et al. (31)	Canada	≈19 ± 2	M		153	−0.08
Knickmeyer et al. (29)^a^	USA/Black	≈1	M/F		31	− 0.08
Mas et al. (34)^a^			M^b^		63	−0.081
Loehlin et al. (30)	Australia	≈14	M		181	− 0.13
Knickmeyer et al. (29)^a^	USA/Asian	≈1	M/F		6	− 0.41
**D_r−l_**						
Knickmeyer et al. (29)^a^	USA/Asian	≈1	M/F		6	0.41
Manning et al. (17)	England	32.6 ± 14.2	M	21.4 ± 2.3	50	0.36***
Hurd et al. (31)	Canada	≈19 ± 2	M		153	0.14
Loehlin et al. (30)	Australia	≈14	M		181	0.10
Knickmeyer et al. (29)^a^	USA/Black	≈1	M/F		30	0.10
Zhang et al. (28)	China	19.9 ± 1.4	F		391	0.055
Knickmeyer et al. (29)^a^	USA/White	≈1	M/F	≈19.7 ± 2.5	105	0.04
Mas et al. (34)^a^			M		70	− 0.021
Zhang et al. (28)	China	19.9 ± 1.4	M		294	− 0.022
Hampson and Sankar (16)	Canada	18.7 ± 1.6	M	22.1 ± 3.1	152	−0.047
Mas et al. (34)^a^			M^b^		63	−0.057
Loehlin et al. (30)	Australia	≈14	F		218	− 0.06
Butovskaya et al. (27)^a^	Tanzania	≈34 ± 13	M	22.5 ± 2.2	107	−0.080

*D_r−l_ indicates right-hand 2D:4D minus left-hand 2D:4D*.

*^a^ Plus personal communication*.

*^b^ Male-to-female transsexuals*.

*p < 0.05. ***p < 0.001.

**Table 3:** **Results of meta-analyses for the relationship between CAGn and 2D:4D**.

	Mean effect size				Random variance		
	ρ	*Z*	*p*	95% CI upper bound	τ	*Q* (df)	*p*
**ALL SAMPLES**							
2D:4D right hand	0.023	1	0.318	0.068	0	10.0(13)	0.696
2D:4D left hand	0.004	0.14	0.888	0.059	0.05	17.2(13)	0.188
D_r−l_	0.027	1	0.320	0.081	0.04	14.5(12)	0.269
**MALE SAMPLES ONLY**							
2D:4D right hand	0.018	0.58	0.564	0.080	0.03	8.8(8)	0.361
2D:4D left hand	−0.005	0.13	0.896	0.070	0.06	11.9(8)	0.155
D_r−l_	0.035	0.818	0.414	0.117	0.08	11.9(7)	0.103

*Estimates for the population correlation ρ and the upper bound of the 95% confidence interval are Pearson correlations*.

## Discussion

Estimates for the population correlations between CAGn and 2D:4D were close to zero and not statistically significant, and even a liberal viewpoint suggests that any relationship is at best very small (largest upper limit for 95% CI in the full data set *r* = 0.08). None or little (and statistically non-significant) random variance was observed. Therefore, sampling error suffices to explain the mixture of significant and non-significant findings and there is no reason to assume that the former meaningfully differ from the latter (35). As mentioned in the method section, the Loehlin et al. (30) and the Knickmeyer et al. (29) samples contained numerous sib-pairs, and this was not reflected in the weighting of these samples in the current analyses. However, Table 1 shows that the results for these samples were either close to the estimates for ρ or else had very small sample sizes and therefore had little impact on ρ estimates in the first place; consequently, somewhat reduced weights for these samples would not have meaningfully altered the outcome of any of the analyses or any conclusions drawn. This is also illustrated by the result of the analysis that excluded mixed-sex samples (i.e., the three Knickmeyer et al. (29) samples).

Overall, the evidence is quite clear then that 2D:4D and CAGn show no substantial relationship. What does this mean for the validity of 2D:4D as a marker of prenatal T effects? Several authors argued that a relationship between CAGn and 2D:4D is to be expected if the latter indeed reflects prenatal T effects (5, 16, 17), the logic being that if variables *A* and *B* correlate, and variables *B* and *C* do as well, then a correlation between *A* and *C* should emerge. However, if *r*_AB_ = 0.40 and *r*_BC_ = 0.20, a reasonable expectation for *r*_AC_ is 0.08, and to differentiate this empirically from the null hypothesis (*r* = 0.00) is difficult.

There is considerable indirect evidence that the link between CAGn and T effects is weak, which is relevant in this context. First, as discussed in the introduction, *in vitro* studies suggest that each additional CAGn repeat lowers T effectiveness by about 2% (cf. Table 1). Thus, a one standard deviation in CAGn [which is about 3.5, Ref. (20)] would result in a T effect change of only about 7% *in vitro*. Changes of this magnitude might only have a moderate effect on 2D:4D: when Berenbaum et al. (4) looked at the effect of a 100% change in T effects by comparing typically developing men with genetic males affected from complete androgen insensitivity syndrome, the group difference in 2D:4D was about *d* = 0.5, which is equivalent to a correlation of *r* = 0.241^1^. Moreover, *in vitro* studies might overestimate the effects of CAGn *in vivo*, where lower androgen sensitivity due to higher CAGn appears to be counterbalanced by higher circulating T levels, at least in adult men (36, 37).

The second line of indirect evidence stems from relationships between CAGn and other T dependent phenotypes. Androgenetic alopecia (patterned hair loss from the scalp), male infertility, polycystic ovary syndrome, and prostate cancer are conditions in the genesis of which T is clearly implicated (38–41). Following the same line of thought that led to the investigation of a potential link between CAGn and 2D:4D (17), numerous studies looked into the link between CAGn and these conditions. Recent meta-analyses of these studies show that evidence for such a link is at best tentative for prostate cancer and absent for the other three (41–43).

Androgens promote muscle growth and therefore affect FFM (44). A similar picture emerges for the relationship between CAGn and FFM. Pertinent studies (45–49) report results for 11 samples (median *N* = 115). Statistically significant results were only obtained for the two male samples in Walsh et al. (49); in either case a *positive* relationship between CAGn and FFM was observed, which runs against expectations.

In a well-controlled intervention study by Woodhouse et al. (44), 61 eugonadal young men received either 25, 50, 125, 300, or 600 mg/week T enanthate treatment for 20 weeks. FFM gains were statistically modeled by T treatment, CAGn, age, initial strength, and other variables. T treatment explained 64% of the variance in FFM gain. The two next best predictors explained another 2 and 1% of variance, respectively, but CAGn was not among them. When T treatment was excluded as a predictor, the best three-variable model explained only 17% of the variance in FFM change, and again CAGn was not among these predictors. In sum then the results of this study do not suggest a sizable negative effect of CAGn on FFM, which is in line with the correlational studies.

Inferences from androgenetic alopecia, male infertility, polycystic ovary syndrome, prostate cancer, and FFM to 2D:4D are tentative because the former concern adult phenotypes whereas the latter is largely determined *in utero* (11, 12). Nonetheless, these domains demonstrate that a T effect on a phenotype does not necessarily mean that CAGn correlates with this phenotype. Therefore, the lack of a substantial link between CAGn and 2D:4D observed here does not necessarily implicate that 2D:4D is not affected by prenatal T.

On the contrary, the absence of a strong relationship between CAGn and 2D:4D makes the interpretation of 2D:4D findings less ambiguous. If 2D:4D was substantially linked to CAGn, the former might reflect AR effectiveness to a considerable degree. Consequently, a given relationship between 2D:4D and the study variable could reflect effects of circulating T, effects of prenatal T, or both. In light of the nil or near-nil relationship between CAGn and 2D:4D it seems less likely that observed correlations between 2D:4D and study variables reflect effects of circulating T instead of prenatal T [see also Ref. (14)].

Hampson and Sankar (16) conceded that 2D:4D tracks large prenatal T differences between groups (e.g., CAIS vs. typically developing individuals) but argued that the lack of CAGn and 2D:4D demonstrates the latter’s inability to reflect finer prenatal T differences within each sex. But I showed here that a sizable relationship between CAGn and 2D:4D may not be expected even when 2D:4D reflects prenatal T effects well. Further, strong relationships between 2D:4D and performance in sports have been consistently shown (50–54), which also speaks against the idea that 2D:4D cannot explain within-sex differences. However, 2D:4D differences tend to be moderate (*d* about 0.4–0.8) between groups that differ strongly in prenatal T effects (4, 9, 10). This suggests that other factors than prenatal steroids strongly affect 2D:4D (55). Indeed, genetic factors unrelated to T have been implied (56, 57). The use of 2D:4D as a marker for prenatal T effects requires that the non-T variance in 2D:4D is not systematically related to the study variable, and at present we know next to nothing about this point. It would therefore be desirable to better understand the non-T variance in 2D:4D, which might open avenues for its statistical control. Further, a systematic review to what extent 2D:4D and other methods that are less accessible but also less controversial [e.g., Ref. (2)] lead to similar conclusions about prenatal T effects on human behavior would appear helpful.

## Conclusion

A meta-analysis of the literature showed no evidence for a relationship between 2D:4D and CAGn. However, closer inspection of the effects of CAGn on T dependent gene activation *in vitro* and of relationships between CAGn and T dependent phenotypic characteristics suggests that normal variability of CAGn has no, very small, or inconsistent effects. Therefore, the observed lack of an association between CAGn and 2D:4D does not undermine the latter’s validity as an indicator of prenatal T effects.

## Conflict of Interest Statement

The author declares that the research was conducted in the absence of any commercial or financial relationships that could be construed as a potential conflict of interest.

## Footnotes

^1^This is because $r=\sqrt{\frac{d^{2}}{d^{2}+4}}.$

## References

[1] Hines M Gender development and the human brain. Annu Rev Neurosci (2011) 34:69–88 10.1146/annurev-neuro-061010-113654 21438685

[2] Cohen-Bendahan CCC van de Beek C Berenbaum SA Prenatal sex hormone effects on child and adult sex-typed behavior: methods and findings. Neurosci Biobehav Rev (2005) 29:353–84 10.1016/j.neubiorev.2004.11.004 15811504

[3] Manning JT Resolving the role of prenatal sex steroids in the development of digit ratio. Proc Natl Acad Sci U S A (2012) 108:16143–4 10.1073/pnas.1113312108 21930921 PMC3182713

[4] Berenbaum SA Bryk KK Nowak N Quigley CA Moffat S Fingers as a marker of prenatal androgen exposure. Endocrinology (2009) 150:4819–22 10.1210/en.2009-0774 19819951 PMC2775980

[5] Breedlove SM Organizational hypothesis: instances of the fingerpost. Endocrinology (2010) 151:4116–22 10.1210/en.2010-0041 20631003 PMC2940503

[6] Dean A Sharpe RM Anogenital distance or digit length ratio as measures of fetal androgen exposure: relationship to male reproductive development and its disorders. J Clin Endocrinol Metab (2013) 98:2230–8 10.1210/jc.2012-4057 23569219

[7] Zheng Z Cohn MJ Developmental basis of sexually dimorphic digit ratios. Proc Natl Acad Sci U S A (2011) 108:16289–94 10.1073/pnas.1108312108 21896736 PMC3182741

[8] Lutchmaya S Baron-Cohen S Raggatt P Knickmeyer R Manning JT 2nd to 4th digit ratios, fetal testosterone and estradiol. Early Hum Dev (2004) 77:23–8 10.1016/j.earlhumdev.2003.12.002 15113628

[9] Hönekopp J Watson S Meta-Analysis of digit ratio 2D:4D shows greater sex difference in the right hand. Am J Hum Biol (2010) 22:619–30 10.1002/ajhb.21054 20737609

[10] Manning JT Kilduff LP Trivers R Digit ratio (2D:4D) in Klinefelter’s syndrome. Andrology (2013) 1:94–9 10.1111/j.2047-2927.2012.00013.x 23258636

[11] Galis F Ten Broek CMA Van Dongen S Wijnaendts LCD Sexual dimorphism in the prenatal digit ratio (2D:4D). Arch Sex Behav (2010) 39:57–62 10.1007/s10508-009-9485-7 19301112 PMC2811245

[12] Malas MA Dogan S Evcil EH Desdicioglu K Fetal development of the hand, digits and digit ratio (2D:4D). Early Hum Dev (2006) 82:469–75 10.1016/j.earlhumdev.2005.12.002 16473482

[13] Trivers R Manning J Jacobson A A longitudinal study of digit ratio (2D:4D) and other finger ratios in Jamaican children. Horm Behav (2006) 49:150–6 10.1016/j.yhbeh.2005.05.023 16040033

[14] Hönekopp J Bartholdt L Beier L Liebert A Second to fourth digit length ratio (2D:4D) and adult sex hormone levels: new data and a meta-analytic review. Psychoneuroendocrinology (2007) 32:313–21 10.1016/j.psyneuen.2007.01.007 17400395

[15] Muller DC Giles GG Manning JT Hopper JL English DR Severi G Second to fourth digit ratio (2D:4D) and concentrations of circulating sex hormones in adulthood. Reprod Biol Endocrinol (2011) 9:57 10.1186/1477-7827-9-57 21521531 PMC3107785

[16] *Hampson E Sankar JS Re-examining the Manning hypothesis: androgen receptor polymorphism and the 2D:4D digit ratio. Evol Hum Behav (2012) 33:557–61 10.1016/j.evolhumbehav.2012.02.003

[17] *Manning JT Bundred PE Newton DJ Flanagan BF The second to fourth digit ratio and variation in the androgen receptor gene. Evol Hum Behav (2003) 24:399–405 10.1016/S1090-5138(03)00052-7

[18] Baculescu N The role of androgen receptor activity mediated by the CAG repeat polymorphism in the pathogenesis of PCOS. J Med Life (2013) 6:18–25 23599814 PMC3624640

[19] Zitzmann M Nieschlag E The CAG repeat polymorphism within the androgen receptor gene and maleness. Int J Androl (2003) 26:76–83 10.1046/j.1365-2605.2003.00393.x 12641825

[20] Buchanan G Yang M Cheong A Harris JM Irvine RA Lambert PF Structural and functional consequences of glutamine tract variation in the androgen receptor. Hum Mol Genet (2004) 13:1677–92 10.1093/hmg/ddh181 15198988

[21] Beilin J Ball EMA Favaloro JM Zajac JD Effect of the androgen receptor CAG repeat polymorphism on transcriptional activity: specificity in prostate and non-prostate cell lines. J Mol Endocrinol (2000) 25:85–96 10.1677/jme.0.0250085 10915221

[22] Chamberlain NL Driver ED Miesfeld RL The length and location of CAG trinucleotide repeats in the androgen receptor N-terminal domain affect transactivation function. Nucleic Acids Res (1994) 22:3181–6 10.1093/nar/22.15.3181 8065934 PMC310294

[23] Ding D Xu L Menon M Reddy JPV Barrack ER Effect of a short CAG (glutamine) repeat on human androgen receptor function. Prostate (2004) 58:23–32 10.1002/pros.10316 14673949

[24] Callewaert L Christiaens V Haelens A Verrijdt G Verhoeven G Claessens F Implications of a polyglutamine tract in the function of the human androgen receptor. Biochem Biophys Res Commun (2003) 306:46–52 10.1016/S0006-291X(03)00902-1 12788064

[25] Irvine RA Ma H Yu MC Ross RK Stallcup MR Coetzee GA Inhibition of p160-mediated coactivation with increasing androgen receptor polyglutamine length. Hum Mol Genet (2000) 9:267–74 10.1093/hmg/9.2.267 10607837

[26] Kazemi-Esfarjani P Trifiro MA Pinsky L Evidence for a repressive function of the long polyglutamine tract in the human androgen receptor: possible pathogenetic relevance for the (CAG)n-expanded neuronopathies. Hum Mol Genet (1995) 4:523–7 10.1093/hmg/4.4.523 7633399

[27] *Butovskaya ML Vasilyev VA Lazebny OE Burkova VN Kulikov AM Mabulla A Aggression, digit ratio, and variation in the androgen receptor, serotonin transporter and dopamine D4 receptor genes in African foragers: the Hadza. Behav Genet (2012) 42:647–62 10.1007/s10519-012-9533-2 22392544

[28] *Zhang C Dang J Pei L Guo M Zhu H Qu L Relationship of 2D:4D finger ratio with androgen receptor CAG and GGN repeat polymorphism. Am J Hum Biol (2013) 25:101–6 10.1002/ajhb.22347 23132707

[29] *Knickmeyer RC Woolson S Hamer RM Konneker T Gilmore JH 2D:4D ratios in the first 2 years of life: stability and relation to testosterone exposure and sensitivity. Horm Behav (2011) 60:256–63 10.1016/j.yhbeh.2011.05.009 21664359 PMC3143220

[30] *Loehlin JC Medland SE Martin NG Is CAG sequence length in the androgen receptor gene correlated with finger-length ratio? Pers Indiv Differ (2012) 52:224–7 10.1016/j.paid.2011.09.009

[31] *Hurd PL Vaillancourt KL Dinsdale NL Aggression, digit ratio and variation in androgen receptor and monoamine oxidase a genes in men. Behav Genet (2010) 41:543–56 10.1007/s10519-010-9404-7 20967566

[32] Schmidt FL Oh IS Hayes TL Fixed- versus random-effects models in meta-analysis: model properties and an empirical comparison of differences in results. Br J Math Stat Psychol (2009) 62:97–128 10.1348/000711007X255327 18001516

[33] *Folland JP McCauley TM Phypers C Hanson B Mastana SS Relationship of 2D:4D finger ratio with muscle strength, testosterone, and androgen receptor CAG repeat genotype. Am J Phys Anthropol (2012) 148:81–7 10.1002/ajpa.22044 22419368

[34] *Mas M Alonso C Hernandez P Fernandez M Gutierrez P Salido E Androgen receptor CAG and GGN polymorphisms and 2D:4D finger ratio in male to female transsexuals. J Sex Med (2009) 6(Suppl 5):419–20

[35] Cumming G Understanding the New Statistics. Effect Sizes, Confidence Intervals, and Meta-Analysis. New York: Routledge (2012).

[36] Crabbe P Bogaert V de Bacquer D Goemaere S Zmierczak H Kaufman JM Part of the interindividual variation in serum testosterone levels in healthy men reflects differences in androgen sensitivity and feedback set point: contribution of the androgen receptor polyglutamine tract polymorphism. J Clin Endocrinol Metab (2007) 92:3604–10 10.1210/jc.2007-0117 17579205

[37] Huhtaniemi IT Pye SR Limer KL Thomson W O’Neill TW Platt H Increased estrogen rather than decreased androgen action is associated with longer androgen receptor CAG repeats. J Clin Endocrinol Metab (2009) 94:277–84 10.1210/jc.2008-0848 18840639

[38] Davis-Dao CA Tuazon ED Sokol RZ Cortessis VK Male Infertility and variation in CAG repeat length in the androgen receptor gene: a meta-analysis. J Clin Endocrinol Metab (2007) 92:4319–26 10.1210/jc.2007-1110 17684052

[39] Morales A Androgen replacement therapy and prostate safety. Eur Urol (2002) 41:113–20 10.1016/S0302-2838(01)00039-2 12074396

[40] Randall VA Physiology and pathophysiology of androgenetic Alopecia. Dermatol Ther (2008) 21:314–28 10.1111/j.1529-8019.2008.00214.x 18844710

[41] Wang R Goodarzi MO Xiong T Wang D Azziz R Zhang H Negative association between androgen receptor gene CAG repeat polymorphism and polycystic ovary syndrome? A systematic review and meta-analysis. Mol Hum Reprod (2012) 18:498–509 10.1093/molehr/gas024 22695532 PMC3457706

[42] Gu M Dong X Zhang X Niu W The CAG repeat polymorphism of androgen receptor gene and prostate cancer: a meta-analysis. Mol Biol Rep (2012) 39:2615–24 10.1007/s11033-011-1014-9 21667251

[43] Zhuo FL Xu W Wang L Wu Y Xu ZL Zhao JY Androgen receptor gene polymorphisms and risk for androgenetic alopecia: a meta-analysis. Clin Exp Dermatol (2012) 37:104–11 10.1111/j.1365-2230.2011.04186.x 21981665

[44] Woodhouse LJ Reisz-Porszasz S Javanbakht M Storer TW Lee M Zerounian H Development of models to predict anabolic response to testosterone administration in healthy young men. Am J Physiol Endocrinol Metab (2003) 284:E1009–17 10.1152/ajpendo.00536.2002 12517741

[45] Campbell BC Gray PB Eisenberg DTA Ellison P Sorenson MD Androgen receptor CAG repeats and body composition among Ariaal men. Int J Androl (2007) 32:140–8 10.1111/j.1365-2605.2007.00825.x 18042182

[46] Guadalupe-Grau A Rodríguez-González FG Dorado C Olmedillas H Fuentes T Pérez-Gómez J Androgen receptor gene polymorphisms lean mass and performance in young men. Br J Sports Med (2011) 45:95–100 10.1136/bjsm.2009.060285 19617210

[47] Lapauw B Goemaere S Crabbe P Kaufman JM Ruige JB Is the effect of testosterone on body composition modulated by the androgen receptor gene CAG repeat polymorphism in elderly men? Eur J Endocrinol (2007) 156:395–401 10.1530/EJE-06-0607 17322500

[48] Voorhoeve PG van Mechelen W Uitterlinden AG Delemarre-van de Waal HA Lamberts SWJ Androgen receptor gene CAG repeat polymorphism in longitudinal height and body composition in children and adolescents. Clin Endocrinol (2011) 74:732–5 10.1111/j.1365-2265.2011.03986.x 21521258

[49] Walsh S Zmuda JM Cauley JA Shea PR Metter EJ Hurley BF Androgen receptor CAG repeat polymorphism is associated with fat-free mass in men. J Appl Physiol (2005) 98:132–7 10.1152/japplphysiol.00537.2004 15377647

[50] Bennett M Manning JT Cook CJ Kilduff LP Digit ratio (2D:4D) and performance in elite rugby players. J Sports Sci (2010) 28:1415–21 10.1080/02640414.2010.510143 20981610

[51] Hönekopp J Schuster M A meta-analysis on 2D:4D and athletic prowess: substantial relationships but neither hand out-predicts the other. Pers Individ Dif (2010) 48:4–10 10.1016/j.paid.2009.08.009

[52] Kilduff LP Cook CJ Manning JT Digit ratio (2D:4D) and performance in male surfers. J Strength Cond Res (2011) 25:3175–80 10.1519/JSC.0b013e318212de8e 21993037

[53] Longman D Stock JT Wells JCK Digit Ratio (2D:4D) and rowing ergometer performance in males and females. Am J Phys Anthropol (2011) 144:337–41 10.1002/ajpa.21407 21302261

[54] Voracek M Reimer B Dressler SG Digit ratio (2D:4D) predicts sporting success among female fencers independent from physical, experience, and personality factors. Scand J Med Sci Sports (2010) 20:853–60 10.1111/j.1600-0838.2009.01031.x 19843265

[55] Forstmeier W Mueller JC Kampenaers B A polymorphism in the oestrogen receptor gene explains covariance between digit ratio and mating behaviour. Proc R Soc Lond B Biol Sci (2010) 277:3353–61 10.1098/rspb.2010.1007 PMC2981936 20534613

[56] Medland SE Zayats T Glaser B Nyholt DR Gordon SD Wright MJ A variant in LIN28B is associated with 2D:4D finger-length ratio, a putative retrospective biomarker of prenatal testosterone exposure. Am J Hum Genet (2010) 86:519–25 10.1016/j.ajhg.2010.02.017 20303062 PMC2850436

[57] Lawrance-Owen AJ Bargary G Bosten JM Goodbourn PT Hogg RE Mollon JD Genetic association suggests that SMOC1 mediates between prenatal sex hormones and digit ratio. Hum Genet (2013) 132:415–21 10.1007/s00439-012-1259-y 23263445

